# *ScannerVision*: Scanner-based image acquisition of medically important arthropods for the development of computer vision and deep learning models

**DOI:** 10.1016/j.crpvbd.2025.100268

**Published:** 2025-05-08

**Authors:** Song-Quan Ong, Nathan Pinoy, Min Hui Lim, Kim Bjerge, Francisco Javier Peris-Felipo, Rob Lind, Jordan P. Cuff, Samantha M. Cook, Toke Thomas Høye

**Affiliations:** a Department of Ecoscience, Aarhus University, C. F. Møllers Allé 8, DK-8000, Aarhus, Denmark; b Institute for Tropical Biology and Conservation, Universiti Malaysia Sabah, Jalan UMS, 88400, Kota Kinabalu, Sabah, Malaysia; c Department of Electrical and Computer Engineering, Aarhus University, Findlandsgade 22, DK-8200, Aarhus N, Denmark; d Arctic Research Centre, Aarhus University, C. F. Møllers Allé 8, DK-8000, Aarhus, Denmark; e Syngenta Crop Protection, Rosentalstrasse 67, 4058, Basel, Switzerland; f Syngenta International Research Centre, Jealott’s Hill, Berkshire, RG42 6EY, UK; g School of Natural and Environmental Sciences, Newcastle University, Newcastle, UK; h Rothamsted Research, Harpenden, Hertfordshire, AL5 2JQ, UK

**Keywords:** Machine learning, Entomology, Biodiversity, Digitization, *Aedes*, *Anopheles*, *Culex*

## Abstract

Computer vision methods offer great potential for rapid image-based identification of medically important arthropod specimens. However, imaging large numbers of specimens is time consuming, and it is difficult to achieve the high image quality required for machine learning models. Conventional imaging methods for identifying and digitizing arthropods, such as insects and spiders, use a stereomicroscope or macro lenses with a camera. This method is challenging due to the narrow field of view, especially when large numbers of arthropods need to be processed. In this paper, we present a high-throughput scanner-based method for capturing images of arthropods that can be used to generate large datasets suitable for training machine learning algorithms for identification. We demonstrate the ability of this approach to image arthropod samples collected with different sampling methods, such as sticky traps (unbaited, in different colors), baited mosquito traps as used by the US Centers for Disease Control and Prevention (CDC) and BioGents-Sentinel (BGS), and UV light traps with a sticky pad. Using different strategies to place the arthropods on a charge-coupled device (CCD) flatbed scanner and optimized settings that balance processing time and image quality, we captured high-resolution images of various arthropods and obtained morphological details with resolution and magnification similar to a stereomicroscope. We validate the method by comparing the performance of three different deep learning models (InceptionV3, ResNet and MobileNetV2) on two different datasets, namely the scanned images from this study and the images captured with a camera of a stereomicroscope. The results show that the performance of the models trained on the two datasets is not significantly different, indicating that the quality of the scanned images is comparable to that of a stereomicroscope.

## Highlights

• A scanner-based method for arthropod imaging is presented that enables the creation of large ML training datasets.
• Optimized scanner settings capture high-resolution images with morphological details comparable to a stereomicroscope.
• The performance of three DL models trained with scanned images and stereomicroscope images did not differ significantly.
• The scanner-based method was of comparable quality to the stereomicroscope when capturing a large image dataset.

## Graphical abstract

**Figure:**
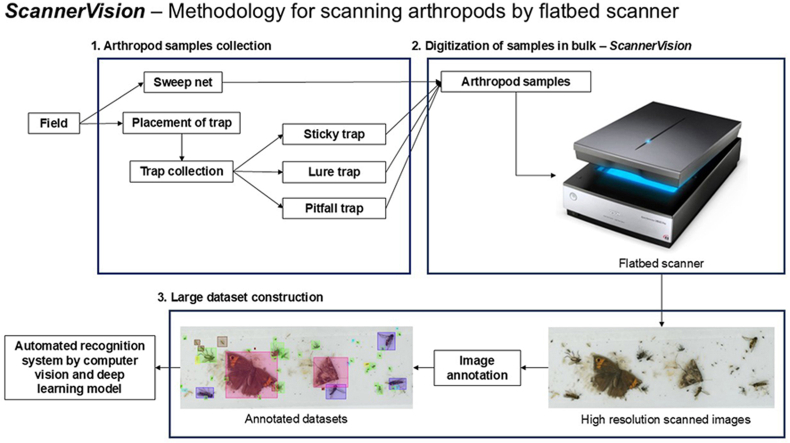

## 1 Introduction

Arthropods, which include insects and arachnids, are the largest and most diverse group of animals on Earth (Thorp, 2009; Mora et al., 2011; Roskov et al., 2021). As pollinators, decomposers and food sources for other species, they play a crucial role in the function and stability of ecosystems (Verma et al., 2023). To study their dynamics and ecological role, arthropod samples are usually collected in the field using active methods, e.g. sweep netting, or passive methods, e.g. pitfall trapping methods, depending on the study objective. The large number of samples, the diversity of species and their complex morphological variation make the identification of arthropods challenging and time consuming. Automated recognition systems using computer vision and deep learning models offer an excellent alternative to support the identification and counting of arthropods in large numbers (Høye et al., 2021; Popescu et al., 2023; Badgujar et al., 2023; Dong et al., 2024; Suresh et al., 2024). However, the size and quality of the training datasets play a crucial role in the performance and generalization of the deep learning models (Popescu et al., 2023). This is particularly true in the context of studying arthropods, where there are very different taxa with a wide variety of morphology. Therefore, good training datasets enable the models to learn the complex morphologies of arthropods for accurate species identification.

Image acquisition should be optimized to produce a high-quality image dataset for arthropod image recognition. This study focuses on techniques for imaging dead arthropods collected in the field using methods such as sticky traps or pitfall traps, where the specimens are killed, and distinguishes these from techniques for imaging living arthropods in their natural habitats. Later, the physical data - the arthropod samples - are converted into digital data - images. Digital photography (with cameras such as the Single Lens Reflective System) and stereomicroscopy are used (Ong et al., 2023; Nolte et al., 2024; Zulzahrin et al., 2024). These methods are usually able to capture fine morphological details of individual specimens but encounter problems when processing a large number of specimens. For example, a Centers for Disease Control and Prevention (CDC) baited light trap placed in a high mosquito infestation area can catch more than 1000 mosquitoes in one day (Ong et al., 2024). Traditionally, the traps would be processed manually by highly trained taxonomists using a stereomicroscope. But taxonomists are in short supply, and results are prone to human error. To aid in standardized, automatic counting and identification, the samples need to be digitized, but this would take a very long time using digital stereomicroscopy methods. This is because the limited field of view of a microscope (the circular area of the viewfinder) usually only allows examination of a relatively limited number of arthropods at a time. The large amount of time required would ultimately hinder model development for automated data collection of arthropods, which would likely further delay downstream data analysis and results, in our example, in a delay in vector-borne disease control.

Charge-coupled device (CCD) flatbed scanners offer an alternative solution to the challenges of large-scale image capture. Buchmann (2011) introduced the flatbed scanner for digitizing moth specimens and emphasized high resolution scanners such as CreoScitex scanners (https://www.scansolutionsonline.com/media/1170/66_eversmart-pro-ii.pdf) that allow effective digitization of moth specimens. They offer uniform illumination, high resolution and consistent focus across the entire image, making them an ideal tool for capturing fine morphological detail without the need for constant adjustments. For example, the imaging system of macroPhor™ Flatbed HSI employs a similar concept using a continuous focus on the target object and eventually creates an image with consistent focus with minimum distortion, compared with other imaging systems such as digital single lens reflective system (DSLR) that rely on a single lens that may create distortions. In addition, Mendez et al. (2018) attempted to use a consumer flatbed scanner with a custom 3D-printed box to image a batch of arthropods stored in liquid at high resolution, but the method can still be improved to capture a larger number of samples. The flatbed scanners can potentially capture many specimens simultaneously, while still capturing important morphological features for identification, such as wing veins, leg segments and body patterns. The consistent image quality of scanners makes them particularly suitable for creating datasets that can be reliably used in machine learning models. In addition, flatbed scanning reduces the variability of lighting and focus that are common in traditional photography, enabling greater standardization of images, and therefore generalization of downstream image-processing methods.

Our aim in this study is to introduce a digitization method capable of processing a large number of arthropods with fine morphological details for the development of deep learning models. We present a method using a consumer-grade, high-resolution CCD scanner with optimized settings for large-scale arthropod imaging. We present different strategies on how to place arthropods on the scanner to achieve image quality comparable with stereomicroscope imaging, when it comes to capturing morphological features such as wing veins, scale patterns on the thorax and legs, antenna type, proboscis and pulps. The method we demonstrate overcomes several limitations of existing imaging methods, including the variable image quality, the high cost of equipment, and the time required to capture individual specimens. With this scanner-based approach, we have shown that it is possible to rapidly digitize many arthropods while maintaining a high standard of image quality across the entire dataset.

## 2 Materials and methods

### 2.1 Sampling of arthropods

To represent a variety of arthropod collection methods, this new method of digitization was conducted at a number of sampling sites using different sampling methods. These were the University Campus at Aarhus University, Denmark (sticky traps), primary forests in North Borneo, Sabah, Malaysia (baited mosquito traps) and urban environments in Singapore (UV light traps with sticky pad). Each site was selected for its arthropod diversity and ongoing biodiversity monitoring with its own research objectives.

The sticky traps, placed at Aarhus University, were used to assess differences in the ability to identify arthropod diversity on traps with different colored backgrounds (Ong and Høye, 2024, 2025). Briefly, two transparent sticky cards (20 × 5 cm, Faicuk, China) were glued to an acrylic plate and later applied in eight different background colors and placed on iron rods at a height of 0.5 m above the ground to capture flying insects. The sticky traps were placed in two consecutive years 2023 and 2024 in summer and early fall (July to September).

Two mechanical traps - Centers for Disease Control and Prevention (CDC style, BGS-Pro, https://eu.biogents.com/bg-pro/) and BioGents-Sentinel (BGS, https://eu.biogents.com/bg-sentinel/) traps, both with artificial human scent and carbon dioxide were used for the baited mosquito traps placed in primary forests in North Borneo, Sabah, Malaysia. We followed the protocol of Ong et al. (2024a); the traps were set in different remote locations in the forest to collect mosquitoes as part of a mosquito-borne disease surveillance project and were run from September 2022 to November 2024. The traps were equipped with a 5 V fan that created suction to drive the arthropods attracted to the trap into an internal netted bag. CDC differed by having an additional LED light source as an attractant.

For the UV light trap with sticky pads placed in urban environments in Singapore, the traps were typically installed to monitor pest flies in food and beverage outlets (Colacci et al., 2020). The trap is equipped with a 20 W lamp with a UVA spectrum of 365 nm (MO-PLIK 399, Italy, https://www.mo-el.com/insect-killers/haccp-mo-stick/mo-plik-399/), which typically attracts a range of different pests such as the house fly (*Musca domestica* L.) as well as various moth species. The sticky pad was white with grid lines and was collected monthly from January to November 2024.

To process the dry arthropod samples from the three sampling methods, the arthropods were killed by freezing at −20 °C. For the sticky cards, the entire card was placed in the freezer for 24 h before scanning. For the CDC and BGS traps, the netted bags were placed in the freezer for 24 h. All samples were placed on an acrylic plate on the scanner for scanning.

### 2.2 Scanner configuration for arthropod imaging

Buchmann (2011) has previously described the specifications of the different scanners and their costs in detail. The scanner we used in this method is a high-resolution CCD flatbed scanner (Epson Perfection V850 Pro), which offers some strong advantages for this application, such as a dual lens system and advanced optics above the CCD sensor that can capture fine morphological details of small specimens. While there are industry-standard scanners with higher resolution used in specimen acquisition (e.g. a range of scanners from CreoScitex), the flatbed scanner we use is much more favorable in terms of cost and convenience. The image quality of the Epson Perfection V850 is better than other flatbed scanners with similar specifications, e.g. the Canon LiDE 400, as it can capture a greater depth of field. Before scanning, the scanner was calibrated with the professional scanning software SilverFast Ai Studio with IT-8 targets (ISO 12641, LaserSoft Imaging AG, Germany) to ensure uniform illumination and color accuracy during the various scanning processes. The scanner (Epson Perfection V850 Pro) was set to professional mode, exposure type: photo, 48-bit color, 1200 dpi, and a high unsharp mask and high contrast were selected for the tone curve. The scanner driver used to operate the scanner requires EPSON Scan Utility v3.9.3.6 to be installed on a computer before the scanner can be operated. Additional tools, such as thin glass to hold the dead arthropod samples collected with pitfall traps, landing nets, and bait traps were used. A thin transparent plastic sheet as a protective layer for the sticky cards that come into contact with the surface of the scanner was used. To keep the surface of the scanner clean, camera cleaning kits, e.g. alcohol-free cleaning agent, blower, brushes, and microfiber cloths, were used. For a brief comparison, Table 1 shows the estimated costs for the devices that can be used for image acquisition at sufficient quality to apply computer vision and deep learning models.

**Table 1:** Comparison of image quality, efficacy, convenience and cost of different types of equipment that can be used to digitize arthropod samples for subsequent use in deep learning model development.

Equipment	Maximum resolution of a single image (dpi)	Ability to process large numbers of arthropods in samples	Degree of convenience in use^a^	Estimated cost (USD)^b^	Example of a device
Digital single-lens reflective system c	72	Low	High	5400	EOS R5 Mark II + RF24-105 mm F4 L IS USM Kit
Stereomicroscope^d^	96	Medium	Low	5200	Olympus SZ-61TR 4K Digital Stereo Microscope
Flatbed scanners	6400	High	Medium	1300	Epson Perfection V850 Pro

a The degree of convenience in use is based on the dimensions, ease of mobility and technical requirements for the device.

b The prices were determined by major retailers on April 30, 2025.

c The digital single-lens reflex system (DSLR) is based on the setting that the images are captured and saved in a compressed JPEG format. The dpi quoted is the equivalent of the field of view generated by an A4-sized paper.

d The estimated cost of different stereomicroscopes may vary from brand to brand.

### 2.3 Placement of the samples on the scanner

The arthropod samples collected by CDC and BGS in Malaysian forests were removed manually from the net bag and placed on a thin glass plate. Forceps were used to arrange the position of the arthropods so that they did not overlap during digitization. For sticky traps used in Denmark, in order to avoid the possible influence of a colored background on the scanning process, a transparent PVC plate was used as a surface for the sticky cards and then attached in front of the background. When the transparent plate was removed from the colored background and scanned entirely on the glass bed of the scanner, it was a transparent plate with sticky cards, without the colored background. The sticky pads used to collect arthropods in urban areas of Singapore were too large to fit whole onto the scanner (card was A3: 29.7 × 42.0 cm in size), so they were cut into three equal parts. Before scanning, a piece of thin, transparent acrylic sheet was used to cover the sticky surface of the card before it was placed on the glass bed of the scanner. For some sticky cards that were attached to a PVC plate, the PVC plate was placed directly on the glass bed and four pieces of cardboard (2 × 2 × 0.5 cm) were used to keep the plate at an even distance from the scanner so that the adhesive surface did not come into contact with the glass surface of the scanner. For optimal imaging, the scanner was cleaned with a microfiber cloth to remove debris.

### 2.4 Scanning process

The scanner software was configured to save the images as high-resolution JPEG files to minimize file size but maintain image resolution for machine learning model development. Notably TIFF format may contain more information about the images, but it is currently not compatible with the machine learning model development pipeline. Therefore, in this study, the files were stored as JPEG with the lowest compression level. Each scan covered a predefined area of the glass bed and allowed many samples to be captured simultaneously. As the sticky card was tagged with metadata, including the trap type, date and collection location, each scanned image could be stored using the metadata and the serial number assigned by the scanner. This metadata served as a unique identification code that was assigned to each specimen and used as a reference during subsequent machine learning training.

### 2.5 Datasets construction

To evaluate the quality and suitability of the images for the development of deep learning models, we developed deep learning models based on image data from two datasets. One was generated by the scanner, the other by a stereomicroscope (Leica MZ16 Stereoscope M125) with an adapted camera mount for a Canon 50D CMOS sensor. Both image datasets derived from the same set of sticky traps (from Denmark and Singapore), and CDC and BGS traps (from Malaysia) i.e. each sample was imaged using both the scanner and the stereomicroscope. The size of the dataset was based on previous studies (Bjerge et al., 2023; Ong et al., 2024); at least 300 images were collected from the scanner or microscope. We collected more than ten orders of arthropods in the field samples, but we focused on the performance of models capable of distinguishing mosquito species (Diptera: Culicidae). Mosquitoes are medically important, and their size and the large quantity in which they were trapped make them a useful group to test the performance of the scanner method. The mosquitoes were assigned to three mosquito genera (classes): *Aedes*, *Anopheles* and *Culex*, using the annotation tool CVAT (https://www.cvat.ai/). The mosquito images were annotated by two entomologists according to the taxonomic keys of Rattanarithikul et al. (2005) and Jeffery et al. (2012) and were cropped as individual images and arranged in a folder according to the classes used for model development.

### 2.6 Comparison of the model developed from the microscope and scanner images

To build the models for automating detection and identification, the mosquito image data were split at random into separate subsets for training (70 %), testing (15 %) and prediction (15 %). After splitting the data, augmentation was performed to avoid using the same original data only for training, testing or prediction. Before developing the deep learning models, a series of data augmentation techniques were performed by rotating all images by 0°, 90°, 180° and 270°. The images were normalized to obtain a range of [0, 1] pixel values and a resolution of 224 × 224. To develop the models, we unfroze the convolutional blocks of the pre-trained convolutional neural networks (CNNs) and re-trained most of the parameters as described in Bjerge et al. (2023, 2024) and Ong et al. (2024b) for three deep learning models: InceptionV3, MobileNetV2 and ResNet. The selection of these hyperparameters was based on Ong et al. (2024b) where the deep learning models were trained using the adaptive learning rate optimization algorithm (ADAM). We used the Keras deep learning framework on an NVIDIA Tesla A100 PCIE GPU platform to train and evaluate the models. Training was performed for 50 epochs and the learning rate ranged from 0.0001 to 0.01 with 32 batches. We implemented early stopping, a regularization technique that halts the training process of a deep learning model development on the validation set when five consecutive epochs show degradation in performance. The performance of these models was evaluated using three metrics: accuracy, precision and recall. The significance of model performance was also compared using the Mann-Whitney *U* test at *P* = 0.05.

## 3 Results

### 3.1 Dataset

The number of images used to develop the model is shown in Table 2. After cropping the individual mosquito images from the original images of the scanner and the stereomicroscope, we obtained a total of 447 cropped images from the stereomicroscope and 436 from the scanner. We performed augmentation by rotating each image 0°, 90°, 180° and 270°, bringing the total number of images to 1788 for the microscope and 1744 for the scanner.

**Table 2:** The number of images of three different mosquito genera used in model development.

	Target classes/genera			Total images
	*Aedes*	*Anopheles*	*Culex*	
Stereomicroscope	800	188	800	1788
Scanner	800	144	800	1744

**Fig. 1:**
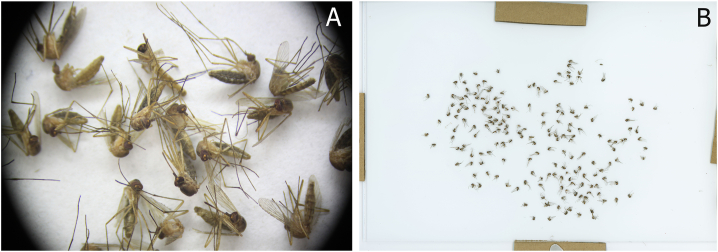
**A** Image of mosquitoes taken with a stereomicroscope (Leica MZ16 Stereoscope M125 stereomicroscope with customized camera mount with Canon 50D CMOS sensor) at 50 × magnification (dimension of image 4752 × 3168 px). **B** Image of sample of trapped mosquitos scanned with the Epson V850 Pro charge-coupled device flatbed scanner according to the methods used in this study (image dimension 10200 × 14039 px).

**Fig. 2:**
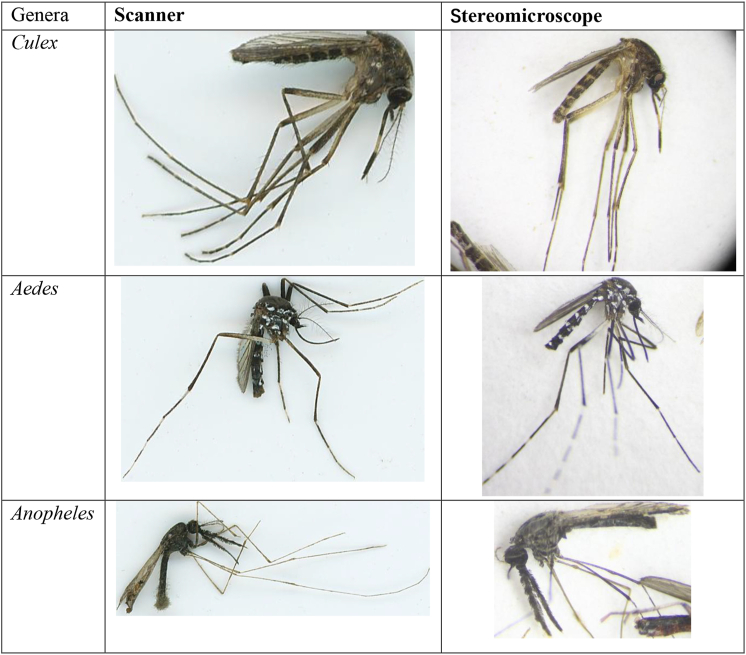
Comparison of images of mosquitoes taken with a flatbed scanner (dimensions from 220 to 650 px) and a stereomicroscope (dimensions from 230 to 800 px) showing the morphology of the specimens in detail.

Images taken with a stereomicroscope (Leica MZ16 Stereoscope M125 stereomicroscope with a customized camera mount for a Canon 50D CMOS sensor) and the scanner are shown in Fig. 1. For the stereomicroscope, despite the lower magnification, the field of view (circular area of view from the eyepiece) can be enlarged, but the number of mosquitoes that can be imaged is still limited (Fig. 1A). In contrast to the stereomicroscope, the scanner, in which all mosquitoes are placed on the glass bed, can capture all collected mosquitoes in one trap sample (Fig. 1B). In addition, Fig. 2 provides a comparison of the magnified scanned images with magnified images taken with the stereomicroscope.

### 3.2 Comparison of model performance between stereomicroscope and scanned images

**Fig. 3:**
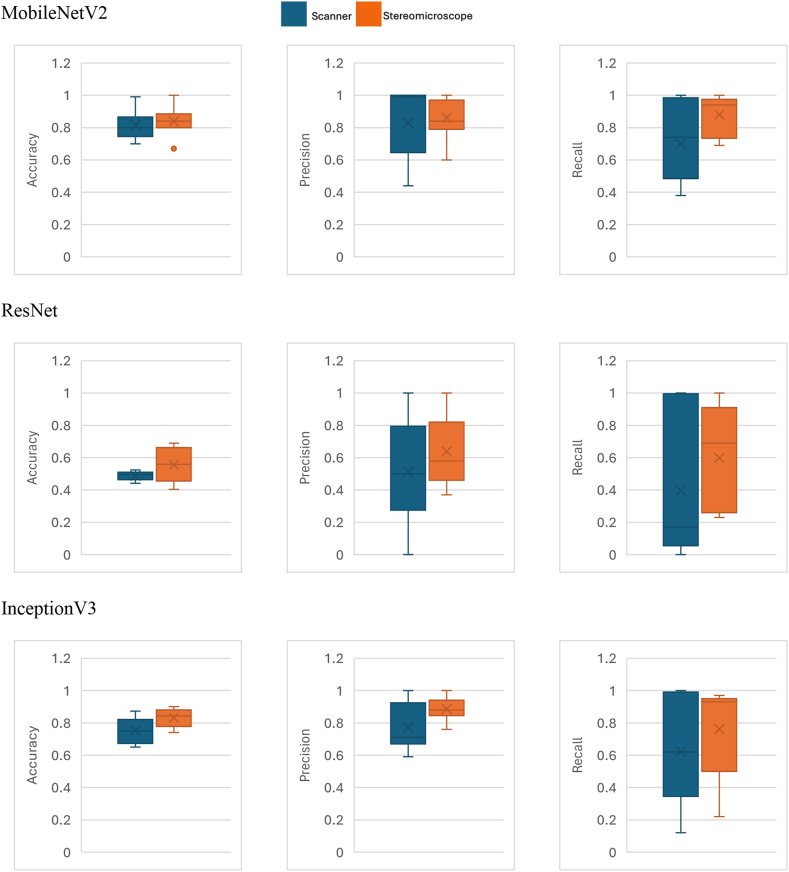
Comparison of accuracy, precision and recall (probabilities that have a maximum value of 1.0) between three deep learning models (MobileNetV2, ResNet and InceptionV3) developed and tested using images produced using a flatbed scanner (*blue bars*) and steriomicroscope with camera attachment (*orange bars*). The box covers the interquartile range, with the median indicated by a horizontal line and the mean indicated by a “ × ”.

Boxplots shown in Fig. 3 provide a comparison of the classification performance of three mosquito genera between the scanner and stereomicroscope images. The performance of the models was similar for both image datasets, although three different deep learning models were used, and the Mann-Whitney *U* test showed no significant difference between the performance of the two datasets (*U* = 9.0, *P* = 0.885; Fig. 3). This indicates that the images acquired with the scanner and stereomicroscope in this study provide a comparable level of performance of the deep learning model despite small variations in the measurements. For more details, Supplementary Fig. S1 and S2 show the training curves - the accuracy curve and the loss function curve for the model performance with scanner and stereomicroscope, respectively, and Supplementary Fig. S3 shows the confusion matrices for the model performance with scanner and stereomicroscope.

## 4 Discussion

We present *ScannerVision*, a method which captures images of a large number of arthropods in a single step, using high resolution and image quality to train fine-grained classification models. The models enabled the classification of arthropod samples from baited light traps into three mosquito genera with an average accuracy of 70.74% for scanned images and 73.65% for stereomicroscope images of three tested models. On the other hand, the performance of the model was consistent with the previous studies by Ong et al. (2024a), in which the MobileNetV2 model was used to classify field-collected *Ae. aegypti*, *Ae. albopictus* and *Cx*. *quinquefasciatus* mosquitoes and achieved an accuracy of 76%. As for the performance of the model, our results generally support the previous studies (Spiesman et al., 2021; Ong and Hamid, 2022; Ong and Høye, 2025) showing that InceptionV3 and MobileNetV2 perform best among other deep learning models such as MnasNet, ResNet, AlexNet, etc., in insect classification. Our study also extends the studies of Pataki et al. (2021) and Ong et al. (2023), which aim to use images of citizen communities to classify *Aedes* mosquitoes, while this study only includes field-collected mosquitoes, so the dataset generated by this method enables a generalizable model. In addition, most of these previous studies captured mosquito images using either a stereomicroscope, a specialized device, a smartphone, or a DSLR, all of which have the limitation of capturing only a limited number of insect vectors in a single image.

**Fig. 4:**
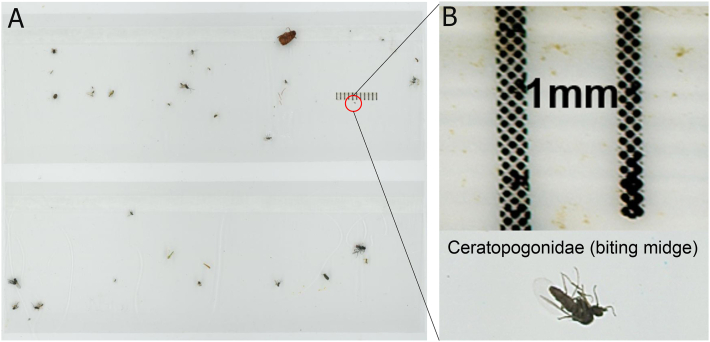
**A** The scanned original image of a sticky card collected from Aarhus University, Denmark. **B** The digital enlargement of a biting midge with a scale-bar (1 mm) at the top. The original image can be viewed *via* the link https://doi.org/10.6084/m9.figshare.28668845.v2.

The resolution achieved by the scanner enabled visibility of fine morphological details of the arthropods, such as the antennae of a biting midge of 1 mm body length (Fig. 4).

**Fig. 5:**
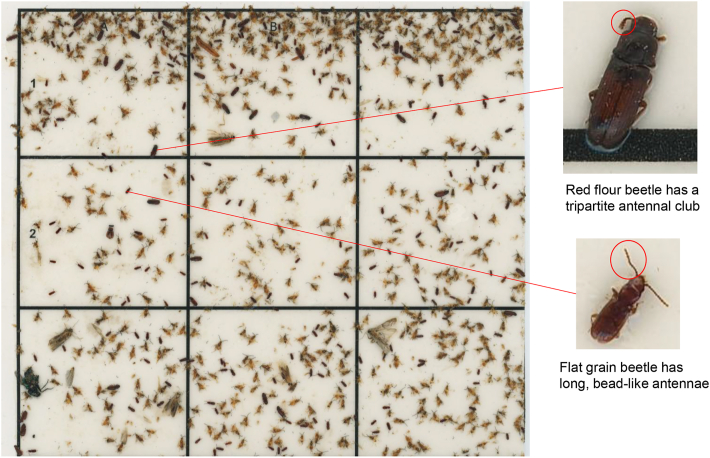
**A** The scanned original image of a UV light trap sticky pad from a warehouse in Singapore. **B** The digital enlargement of the flat grain beetle *Cryptolestes* sp. and the red flour beetle *Tribolium castaneum* with indication (*red circles*) of the morphological distinguishing features that are visible in the image. The original image can be viewed *via* the link https://doi.org/10.6084/m9.figshare.28668845.v2.

The visibility of distinguishing morphological features enabling taxonomical classification from the images produced by the flatbed scanner is illustrated in Fig. 5. The example shows the flat grain beetle *Cryptolestes* sp*.* and the red flour beetle, *Tribolium castaneum* (Herbst); the flat grain beetle has long, bead-like antennae, whereas the red flour beetle has a three-segmented antennal club (https://entnemdept.ufl.edu/creatures/urban/beetles/red_flour_beetle.htm).

Our method extends previous studies that proposed software for counting flies (Yati and Dey, 2011) and ImageJ (Schneider et al., 2012; Parker et al., 2020) commonly used for image analysis, including counting the number of insects, but with the limitation of 8-bit monochrome and one insect species. There are also other imaging techniques to digitize insect specimens producing images of great detail, such as Entomoscope, an open-source photomicroscope for biodiversity discovery, which uses photo stacking to create one sharp image with great focal depth (Wührl et al., 2024). However, Entomoscope does not have the high throughput needed for processing trap samples with hundreds if not thousands of specimens in each.

*ScannerVision* is relatively inexpensive, using a commercially available scanner that allows wide access to this technology compared to expensive cameras and hardware facilities (e.g. stereomicroscope) or industrial scanners (book2net). More importantly, this method could support all existing methods of arthropod sampling, such as pitfall traps and suction trap samples in addition to the sticky traps and baited traps exampled in this study. The portability of the scanner means that it could be used in field stations to generate morphological ‘backup’ data for studies where samples would otherwise have to be destroyed (e.g. for molecular or nutritional analysis) or in situations where it is difficult to transfer physical samples (e.g. sending overseas). For wet samples, e.g. those that have already been stored in alcohol for preservation, a further step may be required to dry the specimens before placing on the scanner.

One of the applications of the method is to support more effective digitization of museum specimens of arthropods, which is currently only possible individually with most methods for arthropods, e.g. pinned specimens (Allan et al., 2019). A large number of captured images could also support higher-level digitization, e.g. by creating a large dataset that could be used to train more robust deep learning algorithms for species classification. As for the next generation of digitization, a standard method for use across the entomology discipline ideally meets the need for open and reproducible data/analysis. There is also much potential for the application of meta-analytical methods if studies use standardized approaches.

*ScannerVision* has, nevertheless, some limitations. First, the image obtained with this method is two-dimensional (2D). While the flatbed scanners provide high-resolution, uniform images, they inherently produce 2D representations of three-dimensional (3D) arthropod specimens. This leads to several challenges. For example, some of the important morphological features of arthropods, such as the scale pattern on the body segmentation, the segments of the legs and the curvature of the wings, are three-dimensional. Flattening the specimen during scanning can obscure or distort these features, especially in complex body structures (e.g. the thorax and abdomen). Incomplete visualization of the specimen is also an inherent constant problem. In some cases, important distinguishing features, such as the underside of an arthropod or fine structures such as the antennae and legs, are not fully visible in a single 2D image. If the animals are positioned in a certain way, important body parts may be obscured, or the visibility of distinguishing features may be limited. To resolve this, multiple scans from different directions (e.g. dorsal, ventral, lateral) may be required, but this increases both the time required and the complexity of image acquisition. For some sample types, such as sticky traps, where the animals are fixed in a certain body position and are difficult to reposition, this is also a major challenge. Combining imaging methods with other biomonitoring technologies, such as molecular approaches, may help to overcome this (Suresh et al., 2024). Secondly, the samples must be stationary, so the method (unlike many other imaging techniques) cannot be used for live samples. Loading and unloading is also an obstacle to automation, unless the method has been extended to a dedicated robotic system [a similar system to the DiversityScanner (Wührl et al., 2022)]. Third, the file size of the high-resolution images generated by the scanner-based system is very large, e.g. the scanned image of this study is in dimensions of 10,200 × 14,039 pixels, file size ranged from 40 to 100 MB per image. While the use of high-resolution scans is essential for capturing the fine morphological details required for accurate arthropod identification, it also presents some challenges. For example, image processing can be problematic, especially when applying pre-processing techniques such as image enhancement, denoising or segmentation. Deep learning models, especially those using convolutional neural networks (CNNs), can be significantly hampered by the need to process large inputs, resulting in slow training times and high memory consumption (Hayakawa and Narihira, 2020). Standard deep learning pipelines often downsize images to reduce the computational load. However, this can compromise the resolution and detail required for accurate classification of arthropods. Alternatively, large images can be split into smaller parts to maintain the original resolution and reduce memory consumption. This can have the disadvantage that objects at the edge of the tiles become only partially visible, and it is more difficult for the CNN to learn. This can be avoided by using overlapping tiles, but this requires larger image datasets. Importantly, if a large proportion of the scanned image does not contain arthropods, it could be discarded. However, this would require either manual processing of the images or coordinated use of specific areas of the scanner’s field of view.

## 5 Conclusions

*ScannerVision* is an improvement in providing high-throughput, high resolution and high detail arthropod images for training deep learning models. The compatibility with existing sampling methods and the ability to create large, standardized datasets can contribute to the standardization of methods in entomology. Although the 2D imaging approach and large file sizes have limitations, these challenges can be mitigated by advances in multi-angle imaging technology, data processing and automation. By enabling open and reproducible workflows in entomology, *ScannerVision* has the potential to standardize imaging procedures and contribute to meta-analytical biodiversity studies.

## Ethical approval

All authors confirm that we have complied with all relevant ethical regulations. For the mosquito collection aspect, this project was approved by the Malaysian Ministry of Health (NMRR ID -23-00934- TOM), the Ethics Committee of University Malaysia Sabah [JKEtika 3/23 (13)] and the Animal Ethics Committee of UMS (AEC 007/2023). We obtained a permit from the Sabah Biodiversity Centre (SaBC) to collect mosquitoes in the forest area of Sabah [JKM/MBS.1000-2/2 JLD.16 (139)]

## CRediT authorship contribution statement

**Song-Quan Ong:** Conceptualization, Methodology, Software, Formal analysis, Investigation, Data Curation, Writing - original draft, Writing - review & editing, Visualization, Project administration. **Nathan Pinoy:** Methodology, Software, Formal analysis, Investigation, Data curation, Project administration. **Min Hui Lim:** Methodology, Software, Formal analysis, Investigation, Data curation, Project administration. **Kim Bjerge:** Methodology, Software, Formal analysis, Writing - review & editing, Supervision. **Francisco Javier Peris-Felipo:** Methodology, Software, Writing - review & editing, Supervision, Project administration, Funding acquisition. **Rob Lind:** Methodology, Software, Writing - review & editing, Supervision, Project administration, Funding acquisition. **Jordan P. Cuff:** Conceptualization, Writing - review & editing. **Samantha M. Cook:** Conceptualization, Writing - review & editing. **Toke Thomas Høye:** Conceptualization, Methodology, Software, Formal analysis, Writing - review & editing, Supervision, Project administration, Funding acquisition.

## Data availability

The data supporting the conclusions of this article are included within the article and its supplementary file. The original images can be viewed *via* the link https://doi.org/10.6084/m9.figshare.28668845.v2.

## Funding

We gratefully acknowledge the support of LivinGro® (Syngenta Crop Protection) by funding the current research. JPC, SMC and TTH were partly funded by the European Union’s Horizon 2020 Research and Innovation Programme as part of the project EcoStack (Grant Agreement no. 773554).

## Declaration of competing interests

The authors declare that they have no known competing financial interests or personal relationships that could have appeared to influence the work reported in this paper.

## Data Availability

The data supporting the conclusions of this article are included within the article and its supplementary file. The original images can be viewed *via* the link https://doi.org/10.6084/m9.figshare.28668845.v2.
