# Supplementary material for: ScannerVision: Scanner-based image acquisition of medically important arthropods for the development of computer vision and deep learning models
Source: Curr Res Parasitol Vector Borne Dis. 2025 May 8;7:100268. doi: 10.1016/j.crpvbd.2025.100268 (PMC12141936; doi:10.1016/j.crpvbd.2025.100268)
Supplement: Multimedia component 1 [file mmc1.pdf]

Supplementary Figure S1. Accuracy plotted against epochs for the training and test split of deep learning models developed and tested using flatbed scanner ‘ScannerVision’ - and stereomicroscope-generated images.

<table>
<tr><td></td><td colspan="2">MobileNetV2</td></tr>
<tr><td>Learning rate</td><td>Scanner</td><td>Stereomicroscope</td></tr>
<tr><td>0.01</td><td>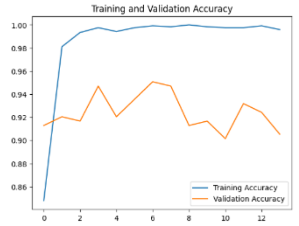
</td><td>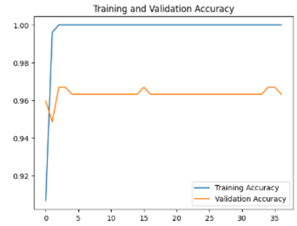
</td></tr>
<tr><td>0.001</td><td>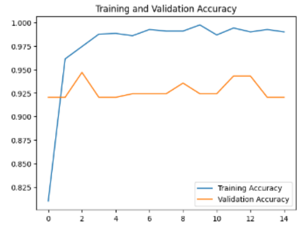
</td><td>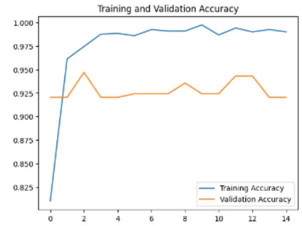
</td></tr>
<tr><td>0.0001</td><td>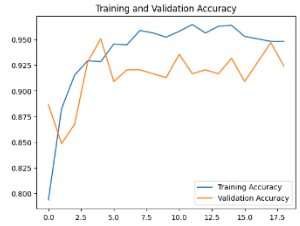
</td><td>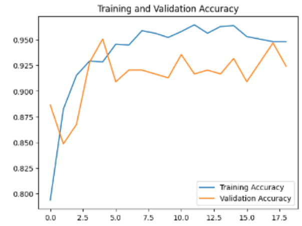
</td></tr>
</table>

<table>
<tr><td></td><td colspan="2">ResNet</td></tr>
<tr><td>Learning rate</td><td>Scanner</td><td>Stereomicroscope</td></tr>
<tr><td>0.01</td><td>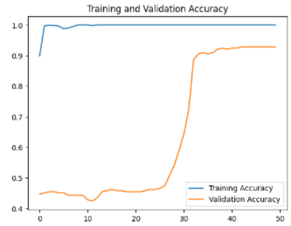
</td><td>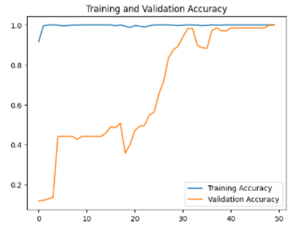
</td></tr>
<tr><td>0.001</td><td>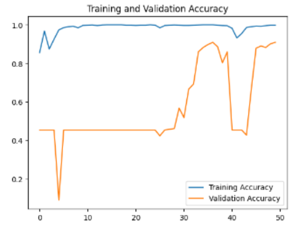
</td><td>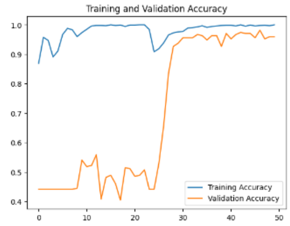
</td></tr>
</table>

<table>
<tr><td>0.0001</td><td>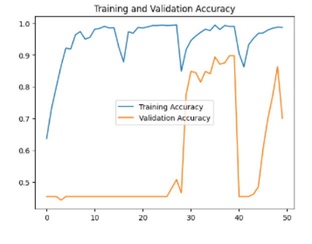
</td><td>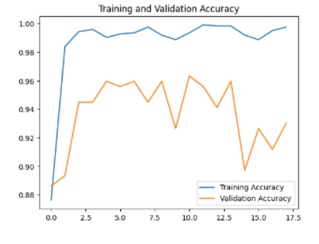
</td></tr>
</table>

<table>
<tr><td></td><td colspan="2">InceptionV3</td></tr>
<tr><td>Learning rate</td><td>Scanner</td><td>Stereomicroscope</td></tr>
<tr><td>0.01</td><td>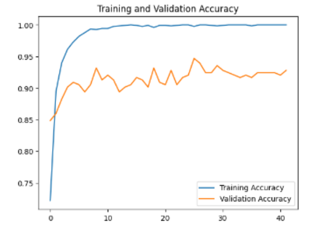
</td><td>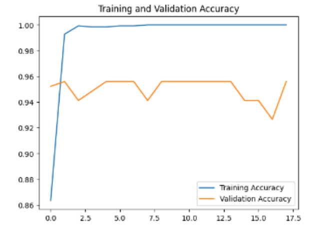
</td></tr>
<tr><td>0.001</td><td>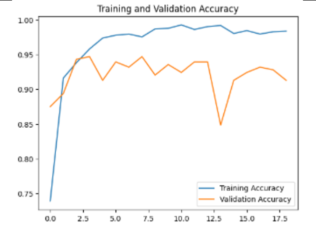
</td><td>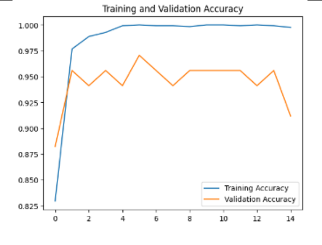
</td></tr>
<tr><td>0.0001</td><td>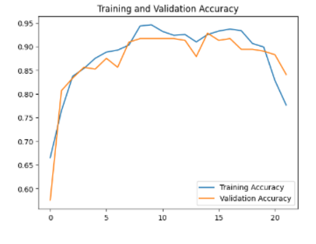
</td><td>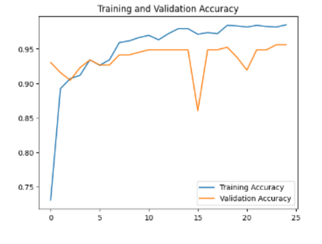
</td></tr>
</table>

Supplementary Figure S2. Loss function plotted against epochs for the training and test split of deep learning models developed and tested using flatbed scanner ScannerVision' - and stereomicroscope-generated images.

	**MobileNetV2**	
**Learning rate**	**Scanner**	**Stereomicroscope**
0.01	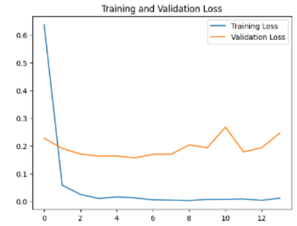	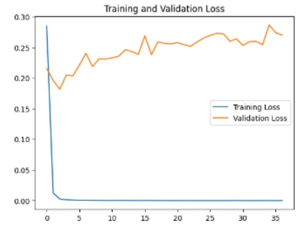
0.001	3 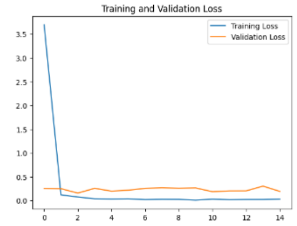	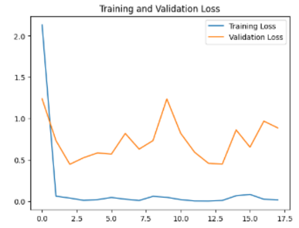
0.0001	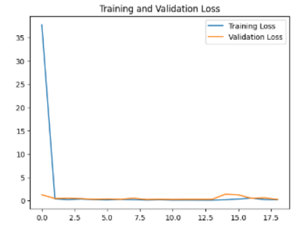	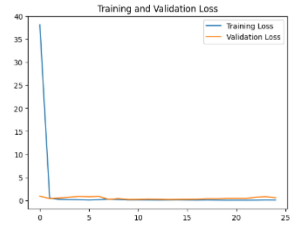

	**ResNet**	
**Learning rate**	**Scanner**	**Stereomicroscope**
0.01	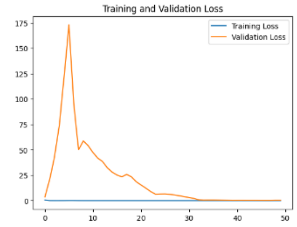	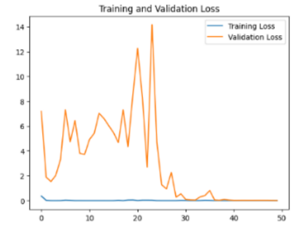
0.001	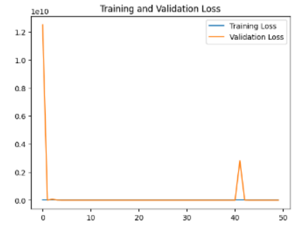	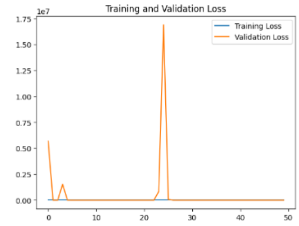

<table>
<tr><td>0.0001</td><td>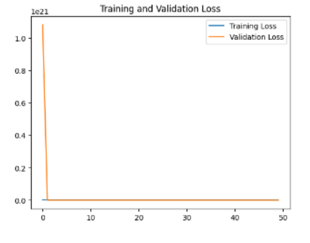
</td><td>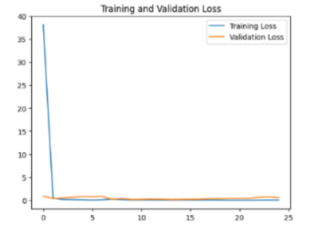
</td></tr>
</table>

<table>
<tr><td></td><td colspan="2">InceptionV3</td></tr>
<tr><td>Learning rate</td><td>Scanner</td><td>Stereomicroscope</td></tr>
<tr><td>0.01</td><td>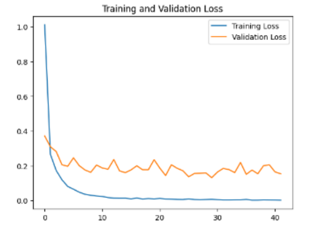
</td><td>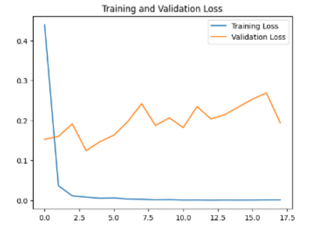
</td></tr>
<tr><td>0.001</td><td>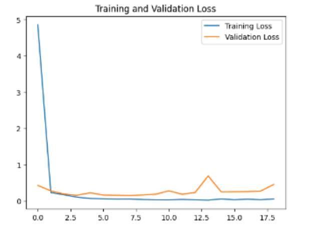
</td><td>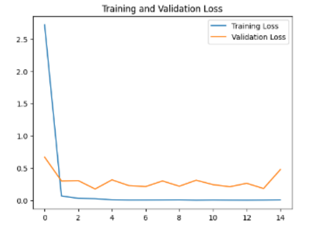
</td></tr>
<tr><td>0.0001</td><td>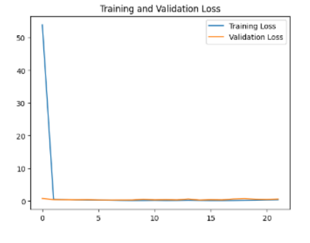
</td><td>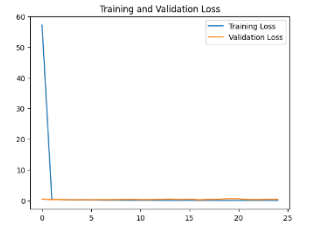
</td></tr>
</table>

Supplementary Figure S3. Confusion matrix for the model performance of the deep learning models developed and tested for flatbed scanner 'ScannerVision' and stereomicroscope images. The x-axis shows the prediction of the deep learning model, the y-axis shows the actual class, the number in the matrices refers to the number of predictions for the actual class, the scale bar shows the density of the prediction (the denser the blue, the more true positives).

	**MobileNetV2**	
**Learning rate**	**Scanner**	**Stereomicroscope**
0.01	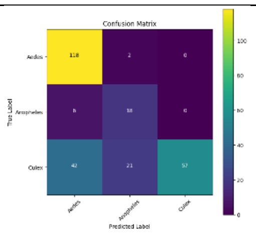	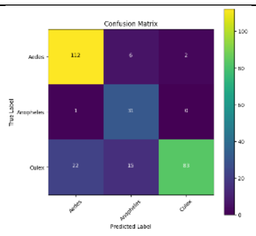
0.001	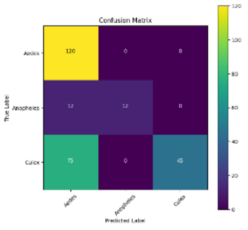	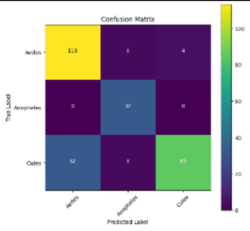
0.0001	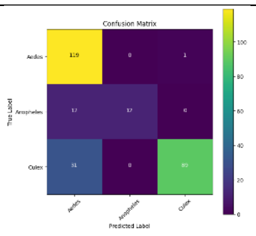	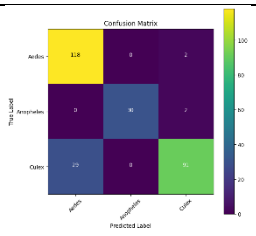

	**ResNet**	
**Learning rate**	**Scanner**	**Stereomicroscope**
0.01	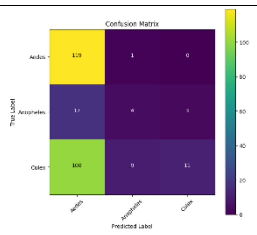	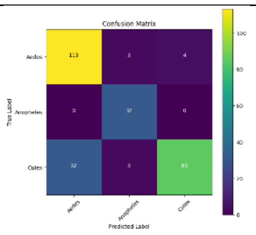
0.001	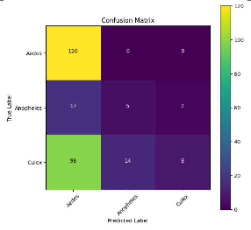	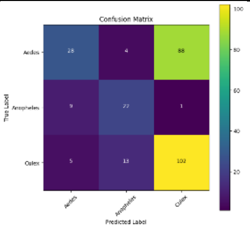

0.0001	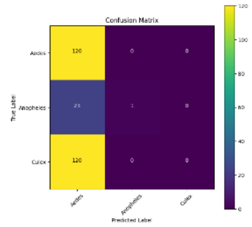	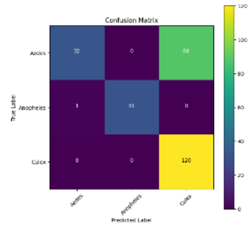

	**InceptionV3**	
**Learning rate**	**Scanner**	**Stereomicroscope**
0.01	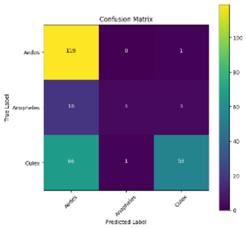	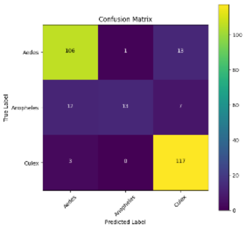
0.001	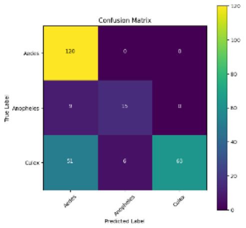	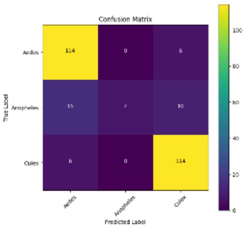
0.0001	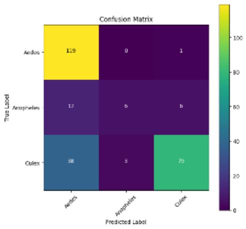	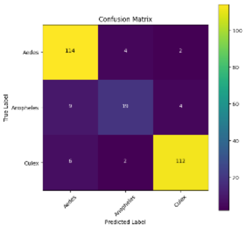
